# A World Allergy Organization (WAO) manifesto for the worldwide approval of biologic therapies for food allergy^☆^

**DOI:** 10.1016/j.waojou.2025.101113

**Published:** 2025-09-20

**Authors:** Alessandro Fiocchi, Bryan Martin, Gary Wing-Kin Wong, Motohiro Ebisawa, Lucia Lo Scalzo, Francesca Galletta, Stefania Arasi, Ignacio J. Ansotegui, Maryam Ali Al-Nesf Al-Mansouri, Sandra N. Gónzalez Díaz, Jonathan A. Bernstein, Elham Hossny, Hiroshi Chantaphakul, Luciana K. Tanno, Tinatin Chikovani, David M. Lang, Luz Fonacier, Hideaki Morita, Mary Beth Fasano, José Antonio Ortega-Martell, Pedro Giavina-Bianchi, Nikolaos G. Papadopoulos, R. Maximiliano Gómez, Mário Morais Almeida

**Affiliations:** a Allergy Diseases Research Area, Pediatric Allergology Unit, Bambino Gesù Children's Hospital IRCCS, Rome, Italy; b Medicine and Pediatrics, The Ohio State University in Columbus, Columbus, OH, USA; c The Chinese University of Hong Kong, Hong Kong, Special Administrative Region of China; d Sagamihara National Hospital, Sagamihara, Japan; e Pediatric Unit, Department of Human Pathology of Adult and Childhood Gaetano Barresi, University of Messina, Messina, Italy; f Hospital Quironsalud Bizkaia, Bilbao, Spain; g Allergy and Immunology Division, Hamad General Hosptial, Hamad Medical Corporation, Doha, Qatar; h Autonomous University of Nuevo León, Hospital Universitario, Centro Regional de Alergia e Inmunología, Monterrey, Mexico; i Division of Immunology/Allergy Section, Department of Internal Medicine, University of Cincinnati College of Medicine, Cincinnati, OH, USA; j Pediatric Allergy, Immunology and Rheumatology Unit, Children's Hospital, Ain Shams University, Cairo, Egypt; k Division of Allergy and Clinical Immunology, Department of Medicine, Faculty of Medicine, Chulalongkorn University, Bangkok Thailand; l Division of Allergy, Department of Pulmonology, Allergy and Thoracic Oncology, University Hospital of Montpellier, Montpellier, France; m Desbrest Institute of Epidemiology and Public Health, UMR UA11 University of Montpellier-INSERM, Montpellier, France; n WHO Collaborating Centre on Scientific Classification Support, Montpellier, France; o Tbilisi State Medical University, Tblisi, Georgia; p Cleveland Clinic, Cleveland, OH, USA; q NYU Grossman Long Island School of Medicine, New York, NY, USA; r National Research Insittute for Child Health and Development, Tokyo, Japan; s University of Iowa Health Care, Iowa City, IA, USA; t Universidad Autónoma del Estado de Hidalgo, Pachuca, Hidalgo, Mexico; u Clinical Immunology and Allergy Division, University of Sao Paulo, Brazil; v Allergy Department, Second Pediatric Clinic, National and Kapodistrian University of Athens, Athens, Greece; w Division of Infection, Immunity and Respiratory Medicine, School of Biological Sciences, The University of Manchester, Manchester, UK; x Faculty of Health Sciences, Catholic University of Salta, Argentina; y Allergy Center, CUF Descobertas Hospital, Lisbon, Portugal; z Bernstein Allergy Group, Bernstein Clinical Research Center, Cincinnati, OH, USA

**Keywords:** Food hypersensitivity, Epidemiology, Anaphylaxis, Biologics, Drug approval process, Biological therapies

## Introduction

Since March 2024, after approval from the United States (US) Food and Drug Administration (FDA) approval, patients allergic to foods in the United States can access biological therapeutic drugs from the age of 1 year onwards to be used in conjunction with food allergen avoidance. This has not yet been adopted in any other country in the world.

Our objective is to highlight that food-related anaphylaxis is a health emergency for which newer therapies have been developed, and which must be made available to patients throughout the world. We recently performed a meta-analysis on randomized controlled trials that assessed current evidence on the impact of biologicals as monotherapy or in combination with oral immunotherapy in patients with food allergy. Other meta-analyses, observational studies, case reports, and systematic reviews on the use of biological therapies in food allergy are also included herein.

Biologicals can increase the threshold of reactivity to foods in food-allergic patients. As a result, the risk of reactions to accidental ingestion (including anaphylactic reactions) to foods is reduced. This translates into improved food-allergy related quality of life for patients with food allergies and their families as well as a potential reduction in the costs incurred by them personally and by the health systems for the management of serious food-related reactions. Although there is no direct evidence, it is very likely that biological therapies substantially reduce morbidity and mortality from food allergies.

We encourage that, as has happened in other cases for the registration of drugs of urgent clinical need, the legislative procedures for the approval of biologicals in countries other than the United States of America, where approval has already been obtained, be streamlined. The availability of biological therapies for the prophylactic management of food allergy represents valuable intervention for these patients, and should be made available on a global level.

## What is a manifesto?

Manifestos are written documents intended to declare aspirations, opinions, intentions, objectives, and purposes, adopted by a movement, a group, or an organization. A manifesto ends up with a call to action to achieve changes in behaviour that result from it. In health care, a manifesto is generally developed to: (1) draw attention to a specific topic, (2) highlight the challenges that await policymakers, health professionals, and patients and (3) foster the adoption of strategies of current scientific relevance. Therefore, it seemed to us that there is no better format to express the position of the World Allergy Organization (WAO) regarding the global availability of biological therapies for food allergy. With this statement, we advocate that these remedies be made available to patients all over the world.

## The US approval of anti-IgE for food allergy

In February 2024, the phase 3 OUtMATCH trial demonstrated the efficacy of an anti-IgE monoclonal in increasing the threshold for allergic reactions to multiple foods and reducing the risk of reactions from accidental exposure, including anaphylaxis.^1^ Immediately after the publication of the study, the US FDA issued an indication for the treatment of IgE-mediated food allergy in adults and children aged 1 year and older, to be used in conjunction with food allergen avoidance. The announcement of the FDA approval caused such a stir that it captured the interest of the major national media. In fact, it is the first and so far the only remedy with this indication worldwide.

## Barriers to worldwide approval of biological therapies for the food allergy indication

The scientific and patient community would expect a rapid extension of this indication to other countries. Eighteen months after the approval, this has not happened. The United States remains the only country that has adopted the food allergy indication for anti-IgE. The main factors delaying the approval worldwide appear to be the following:

1. Regulatory and regional differences in evidence requirements

While the FDA considered the evidence provided by the OUtMATCH trial sufficient, other regulatory agencies (such as the European Medicines Agency and counterparts in Asia-Pacific and Latin America) may require additional or region-specific data before granting approval. This may include longer term safety, efficacy in diverse populations, and real-world effectiveness.

2. Unresolved clinical questions and knowledge gaps

Clinical uncertainties remain regarding optimal patient selection, dosing for patients with high baseline serum total IgE, proper dosage and schedule for patients with food allergy (unclear the role of total IgE in predefining the effective dose and interval of administration), and strategies to monitor or discontinue treatment. Further studies are needed to address some of these gaps, particularly regarding sustained unresponsiveness, disease modification, and use in adults and older populations.^2^^,^^3^

3. Cost-effectiveness and health system considerations

Biological therapies do have high cost, and health technology assessment bodies in many countries require clear evidence of cost-effectiveness and defined patient subgroups who benefit most before recommending widespread adoption.

4. Generalizability of trial data

Most clinical trials have been conducted in predominantly North American, non-Hispanic White pediatric populations, with limited adult data and underrepresentation of other ethnicities and regions. This may limit the ability of some regulatory bodies to extrapolate findings to their populations.

5. Ongoing need for post-marketing surveillance

Regulatory agencies may delay approval pending more extensive post-marketing safety data, especially regarding rare adverse events (although there is evidence from the long-term post-marketing use of anti-IgE, which has been approved for asthma already since 2003) and use in combination with oral immunotherapy^.^

In spite of the challenges and issues discussed above, these barriers should be carefully reconsidered by regulatory bodies throughout the world in the face of a potentially life-threatening disease.

## Food-related anaphylaxis is a persistent emergency

Reports from various regions of the world suggest that the frequency of anaphylactic reaction to foods is increasing worldwide. In Europe, the incidence of food-related anaphylaxis has been rising, with hospital admissions increasing significantly over the past few decades. Data from the United Kingdom (UK) registry indicate an increase in anaphylaxis-related hospitalizations. In that country, between 1998 and 2018 hospital admissions for food-induced anaphylaxis increased from 1.23 to 4.04 per 100,000 population per year, representing an annual increase of 5.7%. This increase was most pronounced in children younger than 15 years, with an annual increase of 6.6%. The anaphylaxis mortality rates in Germany increased from 0.48 in 2009 to 0.59 per 1,000,000 population per year in 2020. In Spain, a significant increase in paediatric patients and food-related anaphylaxis was reported, with a spike of hospitalizations in 2021. Similar alarms come from France, Italy, and Eastern European countries.

In North America, there has been a notable increase in emergency department visits for anaphylaxis, particularly food-related anaphylaxis among children, not only in the United States but also Canada. From 1998 to 1999 to 2011–2012, Australia has also registered a rise in hospital admissions for food-related anaphylaxis, especially in children aged 0–4 years. The rise was from 5.6 to 8.2 per 100,000 population per year, a 1.5-fold increase over 7 years.

In Asia, although data are more heterogeneous and limited by under-reporting, there is evidence suggesting an increasing trend in anaphylaxis incidence. In Singapore, the incidence rate of emergency department visits for childhood anaphylaxis emergency visits doubled from 18.9 to 38.8 per 100,000 person-years between 2015 and 2022. In Korea, the incidence of anaphylaxis nearly doubled from 16.02 in 2008 to 32.19 episodes per 100,000 person-years in 2014. Data from Hong Kong indicate that the incidence of paediatric anaphylaxis showed a rising trend over the past decade (2010–2019), with an estimated incidence of 9.76 per 100,000 person-years. In Africa, while comprehensive data is sparse, there is an emerging concern about the growing prevalence of allergic diseases, including anaphylaxis. In Latin America, food induced anaphylaxis was reported as the second causative agents after drugs, involving 1 out of 3 cases of those severe reactions. Among the provocative agents, milk and egg were identified in paediatric population and shellfish, fruits and fish in adults.

Overall, the global trend indicates an increase in the incidence of anaphylaxis, driven largely by food triggers. While in some countries no increase in mortality has been reported, it is likely that the increase in the number of anaphylactic reactions translates into a greater risk of death related to food ingestion.

## Preventing fatal anaphylaxis

Every death from food-related anaphylaxis is a preventable event. Typically, fatal cases involve people of young age, often otherwise healthy children whose future is abruptly truncated leaving trails of pain, dismay and regrets. Many measures from a personal and social point of view have been put in place, and can be implemented, to avoid these tragedies.

Among the personal measures, prominent are:

- attention to food avoidance;
- education on the recognition of food allergens in complex foods;
- availability of self-injectable adrenaline;
- information on the promptness with which the administration of such a device is necessary;
- current and future availability of self-administered intranasal adrenaline.

The main prevention measures put in place at a collective level include the mandatory labeling of allergenic foods in pre-packaged foods in catering and in common use, and the reduction of the use of highly allergenic foods especially in confined environments, the preparation of legislation for precautionary labelling of allergenic foods (PAL).

The safety net extended by these provisions around food allergy sufferers however, has significant gaps, as none of these provisions is without limitations. To name a few:

- avoidance creates severe limitations on the quality of life of allergy sufferers^4^ and is not without costs;
- education on allergen recognition is difficult to obtain;
- self-injectable adrenaline is not available in all jurisdictions and is sometimes prohibitively expensive;^5^
- adrenaline administration is often delayed, despite extensive educational campaigns to address this knowledge gap.

At a social level, there are more than 100 countries that lack legislation on mandatory food labelling in products intended for consumption. Legislation for PAL is implemented in only a handful of countries.^6^ Legislation for allergenic food labelling in restaurants is in place in European Union Member States, Switzerland, the UK, Norway, Canada, United States, and Philippines.

## Biological therapies are effective in preventing food-related anaphylaxis

This manifesto relies on a recently published systematic review and quantitative metanalyses of interventional studies.^7^ Clinical trial evidence indicates that anti-IgE, compared to placebo, reduces the allergic response to foods, gets desensitisation and increases the threshold of the ingested culprit food(s), independently from the specific food source. Consequently, it minimizes food allergic reactions including anaphylaxis without significant adverse effects. This translates into an improvement in the quality of life measures and a reduction in patients’ and parental burden. Of note, other meta-analyses have documented the efficacy of biologicals on many parameters of food allergy management.

Additionally, case reports, case series, and observational studies concur in indicating valuable real-world evidence for the efficacy and safety of anti-IgE in treating severe food allergies.^8^ Apart from the confirmation of its efficacy in increasing reaction thresholds and improving the quality of life, anti-IgE reduces anaphylaxis risk by 10 times, abolishes the need to avoid trace foods, and allows the free introduction of more than 70% of the originally excluded foods.^9^ The original limit of serum total IgE levels above 1500 kUI/L for commencing anti-IgE in the treatment of food allergy has been exceeded both by the OUtMATCH study, which used a limit of 2000 kUI/L^1^, and by observational studies that report the safe administration of anti-IgE even for patients with much higher starting IgE values.^10^ Therefore, it will no longer be necessary to perform immune apheresis for the introduction to treatment with anti-IgE in such a scenario.^11^ In contrast to the age limitation of 6 years and above for allergic asthma, anti-IgE (omalizumab) for the food allergy indication is FDA approved for patients as young as 12 months of age.^1^ Interestingly, the OUtMATCH study was supported by the National Institutes of Health, which recognized the significant unmet need for effective treatments for patients with multiple food allergies, given the high prevalence, morbidity, and limited therapeutic options currently available. Recent data from the same study indicate that anti-IgE therapy are superior to multi-allergen oral immunotherapy (OIT) in the treatment of multi-food allergy, allowing the safe introduction of a significant number of foods for some time after its suspension.^12^ The long term safety of anti-IgE in pediatric population is witnessed by asthma studies.

## Food allergy guidelines already recommend biological therapies in food allergy

Guidelines and consensus documents are progressively acknowledging the potential role of anti-IgE as adjunctive therapy in selected cases of food allergy. The 2024 GA2LEN ANACARE Consensus Statement supports the use of anti-IgE both as monotherapy and as an adjunct to OIT in selected high-risk patients, particularly those with multiple food allergies, low reaction thresholds, or those who have failed OIT.^13^ A complementary document — the 2024 AAAAI Work Group Report — provides detailed, consensus-based clinical guidance for implementing the treatment in daily practice. Trying to clarify the many questions that arose in the U.S. after the approval of the indication,^2^ it underscores that in the United States anti-IgE may be used even in the absence of prior therapy failure or severe disease, and explicitly recommends against unnecessary access barriers such as additional eligibility restrictions not aligned with the FDA label.^14^ This aligns with previous indications from the 2022 GA2LEN guideline on food allergy, which already acknowledged the potential of anti-IgE as an adjunctive treatment, particularly in facilitating safer OIT in patients with persistent or severe IgE-mediated allergies.^15^ The first guideline to indicate the use of anti-IgE “no ifs or buts” has been the 2022 update of the WAO Diagnosis and Rationale for Action against Cow's Milk Allergy (DRACMA) guidelines for milk OIT.^16^ The 2024 EAACI algorithm for the diagnosis and management of IgE-mediated food allergy also mentions anti-IgE as a promising adjunct in specific context, although it has not yet provided a formal recommendation for their use.^17^ Thus, food allergy guidelines increasingly recognize anti-IgE as a safe and promising therapeutic option for selected patients. Consensus statements now provide a clear signal to regulators and clinicians: anti-IgE is no longer an experimental option, but an emerging standard in the care of complex food allergy.

## The ideal patient for anti-IgE

In the United States, the cost-benefit ratio is debated but could change with the availability of bioequivalent drugs.^18^ The cost-benefit balance must be established country by country, especially where the drug is dispensed with reimbursement by the health system. The severity of food allergy can now be estimated with systems that verify the risk of lethal reactions and the intrinsic severity of the disease, including clinical, quality of life, and economic factors that gravitate around it. We believe that the application of selective severity scoring systems such as the international DEFASE (DEfinition of Food Allergy SEverity) score^19^ could be useful to identify “the first ones in the row” to be prophylactically treated with anti-IgE.

**In summary**: most clinical uncertainties that hinder the worldwide approval of anti-IgE as a treatment for food allergy have been resolved on the basis of current literature.

**We know today** that biological therapies are currently the most effective drugs in preventing food-related anaphylaxis and reducing fatalities due to this condition. Anti-IgE can be safely used at any age after 1 year, even in presence of high total IgE levels, for long periods, in every population and for any food(s).

**We intend** to highlight the need for such drugs to become available to food-allergic patients in all nations of the world. Given the expiration of the drug's patent, cost problems may be partially overcome by the upcoming arrival of bioequivalents. This could facilitate the use of anti-IgE at least in some population groups, but nothing will be possible without approval from the competent regulatory authorities.

**We advocate** to make this therapy available to the greatest possible number of nations in the world.

**In conclusion**: every life badly lived is a slap in the face to human dignity, but the suffering from food allergy can be alleviated today. In all age groups every death is a painful event, but the death of a person with food allergies who is otherwise healthy is even more tragic. A substantial number of deaths from food-related anaphylaxis are now preventable. With this manifesto, the WAO intends to render a service to patients around the world by calling the community to a common effort.

## Abbreviations

AAAAI, American Academy of Allergy, Asthma and Immunology; ANACARE, Anaphylaxis Centers of Reference and Excellence; DRACMA, Diagnosis and Rationale for Action against Cow's Milk Allergy; EAACI, European Academy of Allergy, Asthma and Clinical Immunology; FDA, Food and Drug Administration; GA2LEN, Global Allergy and Asthma Excellence Network; OutMATCH, Omalizumab as Monotherapy and as Adjunct Therapy to CHildren study; OIT, Oral immunotherapy; PAL, Precautionary Allergen Labeling; WAO, World Allergy Organization.

## Funding

Not applicable.

## Availability of data and materials

All data supporting the statements are published.

## Author contributions

All authors contributed to data interpretation and manuscript revision. AF drafted the initial version. BM, GWKW, ME, LLS, FG, SA, IJA and MMA revised and harmonized it. All authors reviewed and approved the final version of the manuscript.

## Ethics approval

Not applicable.

## Consent for publication

All authors approved the final version and its submission.

## Disclosure statement regarding use of generative artificial intelligence (AI) and AI-assisted technologies

Nothing to disclose.

## Declaration of competing interest

AF has received Speaker honoraria and advisory panel consultancy outside the submitted work for Nutricia, Abbott, Danone, Stallergenes, DBV, Novartis. Funded research (Institution) from Sanofi, Novartis, Ferrero, DBV, GSK, Astrazeneca, Hipp GmBDH, Humana SpA.

IJA reports personal fees from Bayer, Bial, Cipla, Eurodrug, Faes Farma, Gebro, Glenmark, Opella, Menarini, MSD, Roxall and Sanofi outside the submitted work.

SA declares that she has participated as an advisory board member, and/or consultant, and/or speaker/chair at scientific meetings for Aimmune, DBV, Ferrero, Mabylon, Novartis, Stallergenes Greer, Thermo Fisher Scientific and Ulrich outside the submitted work. Funded research (Institution) from Italian Minister of Health and Italian Minister of Education.

BM declares Speaker honorarium and advisory panel consultancy from Sanofi and Merck.

ME reports personal fees from Viatris and SAB of ARS-Pharmaceuticals, Novartis, and Sanofi outside the submitted work.

PGB declares speaker honoraria from Novartis.

DML reports that he has served as a consultant for, carried out clinical research with, and/or has received honoraria from: AstraZeneca, Celldex, Genentech, Novartis, Sanofi-Regeneron.

LF has participated in the advisory board and/or consultant/speaker or received research grant paid to NYU Grossman Long Island School of Medicine from Regeneron, Sanofi, AstraZeneca, Dermavant, Blueprint.

NGP reports that he has served as a consultant for, carried out clinical research with, received grants from, and/or has received honoraria from: Abbott Nutrition, Berlin – Chemie AG, Danone, DBV Technologies, GSK, HAL Allergy, Hyproca Nutrition, MED Maps SRL, Menarini, Nestle Nutrition Institute, Numil, Regneron, Vianex, Vibrant America.

JB reports grants or contracts from Allakos, Areteia, AstraZeneca, Astria, Celldex, Evoimmune, Genentech, Incyte/Escient, Intellia, IONIS, Jasper, Novartis, Pharvaris, Springer, TLL, and Yuhan; royalties from Amgen, Cogent Pharmaceuticals, CRC Press, and UpToDate; consulting fees from Advanced Medical, Areteia, AstraZeneca, Astria, Best Doctor/Teledoc, BMJ, Celldex, DBV, Eli Lilly, Elsevier, Escient, Evoimmune, Genentech, Guidepoint, GUF, Imedics, Incyte/Escient, INEOS, Intellia, IONIS, Jasper, Kenvue, Novartis, Pharvaris, Telios, TEVA, and Yuhan; and speaking fees from Jasper.

HM reports consulting/advisory board agreements with Sanofi, LEO Pharma, and Otsuka Pharmaceutical Co., Ltd.

MMA reports personal fees from Astrazeneca, Bayer, Eurodrugs, FAES Farma, GSK, Menarini, MSD, OM Pharma, Sanofi and Viatris, outside the submitted work.

All other authors have no conflict of interest within the scope of the submitted work.
